# When Support Hurts: Re-Examining the Cyberbullying Victimization–Mental Health Relationship Among University Students in Saudi Arabia

**DOI:** 10.3390/ejihpe16010007

**Published:** 2026-01-01

**Authors:** Ibrahim A. Elshaer, Alaa M. S. Azazz, Chokri Kooli, Mansour Alyahya

**Affiliations:** 1 Department of Management, School of Business, King Faisal University, Al-Ahsa 31982, Saudi Arabia; 2 Social Studies Department, College of Art, King Faisal University, Al-Ahsa 31982, Saudi Arabia; 3 Graduate School of Public and International Affairs, Faculty of Social Sciences, Social Sciences Building 120 University Private, Room 6005, Ottawa, ON K1N 6N5, Canada; 4 Royal Military College of Canada, Department of Management, Faculty of Social Sciences, Kingston, ON K7K 7B4, Canada

**Keywords:** family support, cyberbullying, victimization, mental health, depression, stress, anxiety, KSA university students

## Abstract

Cyberbullying generally reveals two leading players: the attacker side (perpetrator) and the victim side; each side has its distinctive social and psychological dynamics. In most prior empirical studies, the victim side is pivotal, as it bears the direct psychological and emotional consequences of online aggression. Recently, cyberbullying victimisation has been elevated as a main psychological concern among university students. Nevertheless, the moderating role of family support remained untested, particularly in a collectivist cultural context such as the Kingdom of Saudi Arabia (KSA). This study tested the impacts of cyberbullying victimisation on mental health consequences (anxiety, stress, and depression) among KSA university students. The study further tested family support as a moderator in these relationships. Data was collected from 650 students employing a self-structured survey. The data obtained was analyzed using “Partial Least Squares Structural Equation Modelling” (PLS-SEM). The findings revealed that cyberbullying-victimization can significantly raise students’ anxiety, stress, and depressive signs, supporting its place as a critical psychological risk factor. Contrary to the “traditional stress-buffering theory”, family support failed to alleviate the influence of cyberbullying-victimization on anxiety and stress, and unexpectedly, higher levels of family support were related to higher depressive levels, suggesting a reverse-buffering impact. These results highlighted the complicated relationships between family support and emotional outcomes in the context of digital threats. The study stressed the urgent need for culturally delicate mediations, such as training sessions for digital resilience, and colleague-based supportive systems to successfully deal with the mental health consequences of cybervictimization.

## 1. Introduction

The rapid incorporation of AI technologies into university education has transformed how higher education students connect, collaborate, and build social relationships (Barakina et al., 2021; Chan, 2023; Kazimova et al., 2025; Singh & Hiran, 2022). Social online networks such as Instagram, WhatsApp, X, TikTok, and other university learning platforms have become a predominant part of students’ regular social and academic lives (Backer & Awad, 2025; Mills et al., 2024). Nonetheless, in addition to these advantages, the digital environment has also shown some harmful impacts. Noticeably, the concern of cyberbullying, which has surfaced as a main mental health concern impacting university students (Slonje et al., 2013).

Cyberbullying among university student populations is distinct from that recognised among adolescents and non-student adults, signalling differences in developmental stage, social context, and institutional environment. Unlike non-student adults and adolescents, where cyberbullying is regularly embedded in the school context and influenced by parental monitoring and teacher involvement (Marciano et al., 2020; Snakenborg et al., 2011), university students are generally more autonomous, entrepreneurship-oriented (Mohamed et al., 2023; Shabeeb Ali et al., 2023), and more digitally engaged (Vaill et al., 2020). In the university context, cyberbullying often occurs on educationally and socially embedded platforms, such as course group chats, teaching management systems, and social networking platforms (SNPs), closely linked to academic achievements and emerging academic identity (Faucher et al., 2014). These settings exhibit distinctive dominance dynamics that affect academic competition, colleague evaluation, and reputational issues, potentially strengthening the psychological impact of online victimisation (Schenk & Fremouw, 2012). Consequently, cyberbullying between university students should be conceptualized as a contextually and developmentally specific phenomenon rather than a direct extension of adolescent cyberbullying, with distinct forms, mechanisms, and mental health consequences.

Cyberbullying is conceptualized as planned, and repeated aggression conducted through digital online communication platforms, incorporating threats, harassment, degradation, impersonation, or the publishing of harmful content online (Kowalski et al., 2014; Piotrowski & Watt, 2024). Different from traditional bullying, cyberbullying happens constantly outside the physical boundaries, making its effects more enduring and pervasive. Some previous evidence revealed that cyberbullying among higher education students is rising at a worrying rate. Multinational research across 14 different countries argued that almost one in three higher education students had been subjected to cyberbullying in the previous year (Guo et al., 2025). A more updated meta-analysis conducted in 2024 further proved that cyberbullying victimization is greatly correlated with symptoms of depression, anxiety, stress, and suicidal intention among higher education students (Zhu et al., 2024). University students frequently face academic pressures, identity discovery, career ambiguity, and heightened autonomy; these factors can deepen their exposure to the emotional outcomes of harassment in online networks (Chen et al., 2024).

Despite the increasing literature outlining the direct effects of cyberbullying, less research has explored the protective elements that can alleviate these emotional harms. One such element is family support, which plays a major role in psychological stability, even during emerging adulthood. Although higher education students are regularly physically distant from their families, research has consistently shown that family support is a main source of resilience, coping, and well-being (Barrera & Li, 1996; Dorrance Hall & Scharp, 2021). According to the Stress-Buffering Hypothesis (Cohen & Wills, 1985), high levels of family and social support can reduce the adverse psychological effects of stressful events by providing emotional support, assistance, and a sense of security. Updated empirical research confirmed this mechanism as family support can significantly moderate the path from digital stressors to mental health consequences among young adults (Chen et al., 2024; Y. Liu et al., 2025). However, very limited research has tested family support as a moderator of cyberbullying to mental health precisely within the university context, specifically in non-Western contexts where family bonds are often sturdier and more central in determining psychological wellbeing. This gap emphasised the urgent need for in-depth exploration of interpersonal factors that can alleviate the harmful effects of cyber-aggression among university students. Accordingly, this paper aimed to (1) explore the effect of victimization on the mental health of university students and (2) examine if family support can moderate these effects. Testing this moderate role is critical for developing proactive programs, healing university counseling services, improving family commitment efforts, and establishing evidence-based interventions.

## 2. Theoretical Foundations and Hypotheses Formulation

Cybervictimization (the victim side of cyberbullying) has arisen as one of the main prevalent digital threats impacting the well-being of university students (X. Liu et al., 2025; Sorrentino et al., 2023). Unlike traditional bullying, cyberbullying is more intrusive, iterative, and psychologically damaging as it can exceed the physical boundaries of physical bullying, functioning anonymously and occurring in multiple online networks (Kowalski et al., 2023). Higher education students are remarkably vulnerable, as they depend heavily on digital interaction for academic and social purposes, worsening their exposure to online harassment (Zhu et al., 2024). This continuing exposure can foster substantial mental health consequences. The General Strain Theory (GST) can offer a strong lens for recognizing the harmful outcomes of cybervictimization. As per Agnew (Agnew, 2007), straining conditions that are recognised as unreasonable or challenging to cope with lead to adverse emotional consequences and maladaptive outcomes. Cybervictimization has such a strain as its instability, probable anonymity, and public visibility strengthen psychological harm. Some empirical evidence confirmed the GST’s assumption, revealing that recurrent cybervictimization can generate strong psychological strain that can contribute directly to depression symptoms, psychological instability, and stress disorders among adults (Barlett et al., 2021; Varela et al., 2022). Additionally, according to Agnew’s framework (Agnew, 2007), experiences with recurrent ccybervictimizationmay create negative emotions such as frustration, anger, and psychological suffering, which can increase the probability of maladaptive coping strategies. These coping strategies might include internalizing outcomes (e.g., depression, anxiety, and stress symptoms) as well as externalizing attitudes, such as retaliating or executing cyberbullying against others as a form of revenge or strain release.

Likewise, the “Affective Events Theory” (AET) posits that adverse interpersonal circumstances trigger emotional responses that can accumulate to influence long-term emotional states (Weiss & Cropanzano, 1996). Cybervictimization fits this assumption because it is frequently carried out swiftly, unexpectedly, and repeatedly across digital networks. The psychological instability triggered by digital aggression, such as humiliation, fear, or fury, can gradually evolve into more dangerous mental health consequences if the experience persists over a longer period of time (Malik et al., 2021). AET has been widely applied to digital interaction contexts, supporting that online and digital adverse events can significantly reinforce stress and depressive affect among higher education students (Wright, 2024). Empirical evidence further highlights the harmful impacts of cybervictimization. Meta-analyses revealed that cybervictimization is greatly connected with a greater increase in signs of stress, anxiety, and depression, as compared to traditional bullying (Kowalski et al., 2014; Vieta-Piferrer et al., 2024). University students who are subjected to cybervictimization often show invasive thoughts, low self-pride, social detachment, academic problems, and psychological exhaustion. Furthermore, because cybervictimization repeatedly occurs in digital spaces, victims may be subjected to persistent reputational damage and severe psychological strain (Wright, 2017). These adverse impacts are amplified when university students lack sufficient social support or when cybervictimization co-occurs with university workload stressors (Agnew, 2007).

### Cyberbullying-Victimization and Mental Health

Cyberbullying victimization (cybervictimization) is related to anxiety symptoms among university students. Online violence can create a lasting sense of ambiguity, worry, and loss of control, which are the main psychological antecedents of anxiety symptoms (X. Liu et al., 2025). The borderless nature of cybervictimization widens students’ perceptions of threat and sustains ongoing concern about future threats. The invisible nature of the attacker (offenders) worsens this psychological distress (Zhu et al., 2024). When offenders are invisible, victims may be subjected to digital paranoia (the continuous inquiry about who might threaten them) or even broadcast harmful content unintentionally (Varela et al., 2022). This uncertain environment can intensify a protective anxiety attitude (Al-Sabi et al., 2024). University students systematically become fearful of checking their mobile phones, posting content, or contributing to online academic conversations, dreading further harassment or humiliation (Wright, 2015).

Emerging findings showed that cyberbullying victimization can significantly disrupt psychological regulation activities, causing a maladaptive avoidance and coping attitude. Liu et al. (Y. Liu et al., 2025) argued that university students who experienced cybervictimization showed higher levels of anxiety, withdrawal from social interactions, and fear of adverse assessments. Additionally, the social assessment of cyberbullying can make higher education students specifically vulnerable because colleagues’ acceptance plays a major role in academic involvement and identity progress during adulthood (Vieta-Piferrer et al., 2024). As a consequence, cybervictimization damages students’ social support, fuels the feelings of isolation, and reinforces social anxiety in online and offline settings. Therefore, the following hypothesis is introduced:

**H1.**  *Cyberbullying victimization is positively correlated with anxiety (as a mental health disorder).*

Cybervictimization subjects university students to a broad range of unmanageable online hazards, involving hostile posts, public humiliation, and the sharing of private data (Chen et al., 2024; Sorrentino et al., 2023). These online threats regularly occur in substantially visible online environments, strengthening the psychological damage by threatening the victim’s reputation and identity among colleagues (Kowalski et al., 2014). Since online subjects can be promptly published and constantly archived, victims are repeatedly subjected to a deep loss of control, strengthening the sense of humiliation, incapacity, and social helplessness (Zhu et al., 2024). Furthermore, the 24/7 availability of digital platforms signals that cybervictimization is not constrained to university hours or physical contexts (Slonje et al., 2013). Higher education students are regularly connected through smartphones, learning platforms, and social networking sites, creating a context in which provocation can occur at any time without warning (Al-Amer et al., 2025; Alghamdi et al., 2025). This persistent experience can contribute to ongoing hypervigilance, as victims spontaneously examine their devices for further threats, resist disengaging from online windows, and expect further threats (Chen et al., 2024). Over time, this increased state of attentiveness leads to expressive exhaustion, sleep disorders, problems with concentration, and degraded psychological resistance.

From a psycho-physiological standpoint, cybervictimization can trigger the prolonged “fight-or-flight” reaction, triggering cortisol-driven stress paths related to the feeling of fear, being scared, and to cognitive disturbance (Cao et al., 2024; Snakenborg et al., 2011). Previous research has indicated that this chronic triggering weakens executive performance, decision-making processes, and academic achievement, repeatedly resulting in a high level of absenteeism among higher education students (Al-Amer et al., 2025). An increasing body of knowledge and evidence also indicates that university students subjected to recurrent cybervictimization are developing maladaptive attitudes, such as escapism and social life withdrawal, which further strengthen stress levels (Vieta-Piferrer et al., 2024). Based on the previous evidence, the following hypothesis is introduced:

**H2.**  *Cyberbullying victimization is positively correlated with stress (as a mental health disorder).*

Depression embodies one of the main reflective and devastating psychological outcomes of cybervictimization, especially among higher education students managing the pressures of social, academic, and personal progress (Al-Amer et al., 2025; Alghamdi et al., 2025). Unlike the temporary psychological distress, depression reproduces a continued and prevalent weakening in psychological functioning, and cybervictimization has been recurrently recognized as a main predictor of depressive symptoms in adults (Snakenborg et al., 2011). Victims repeatedly report a sense of futility, constant sadness, sensitive detachment, and a loss of motivation, all of which inhibit daily academic achievement and quality of life (Abubakar et al., 2016). The digital feature of cybervictimization intensifies its depressive effects. Online aggression is often public, with observers such as classmates or even a global audience, which can increase feelings of degradation and social conquest (Piotrowski & Watt, 2024). It is also long-lasting, as destructive content, pictures, or posts might stay online forever or be reshared continually, causing an iterative cycle of psychological depression (Zhu et al., 2024).

Increasing evidence reveals that the emotional strain produced by cybervictimization echoes the brain’s reactions to physical hurt. Chronic experience of online provocation can weaken cognitive abilities such as mind concentration and decision-making processes, leading to depressive symptoms (Varela et al., 2022). Furthermore, cybervictimization often co-occurs with isolation and the academic strain in university students’ lives, which increases the risk of fostering severe depressive symptoms (Wright, 2024). Meta-analytic evidence supported that cybervictimization leads to a higher level of suicidal intentions than conventional bullying due to its extensive influence and the inability of victims to avoid the digital context (Lee et al., 2025). University students who are subjected to continual digital threats often feel blocked, powerless, and psychologically overwhelmed, reinforcing a sense of vulnerability to depression (Alghamdi et al., 2025). Therefore, the following hypothesis is introduced:

**H3.**  *Cyberbullying victimization is positively related to depression (as a mental health disorder).*

Family support has widely been tested as a main protective element in alleviating the harmful psychological outcomes of stressful life conditions, including cybervictimization. Based on the “Stress-Buffering Hypothesis” (Cohen & Wills, 1985), the theory posits that people with strong supportive bonds to family members can better cope with emotional burdens. Family support repeatedly provides emotional encouragement, informational control, and assistance, which can help victims deal with threatening events, adjust emotional reactions, and sustain emotional stability despite external stressors (Dınn & Caldwell-harrıs, 2016; Horwitz et al., 2015). Social support has long been recognized as a critical determinant of mental health and well-being. Additionally, according to Cohen and Wills (Cohen & Wills, 1985), social support can be operated through two main complementary instruments: (1) the buffering hypothesis and (2) the direct effect hypothesis. While the first buffering hypothesis argues that social support can protect people from the harmful psychological consequences of stress by acting as a moderator in the relationship between stressors and mental health consequences, the second direct effect hypothesis posits that social support can exert a positive impact on well-being regardless of the stress levels. University students who are subjected to cybervictimization might hide the psychological harm, feel embarrassed, or even isolate themselves, believing they should cope alone. Family support can stop this isolation by promoting two-way communication and reassuring disclosure (Guo et al., 2025). Such settings foster a sense of belonging and minimize the helplessness that often arises after repeated online aggression. Some empirical evidence confirmed that family support can play a significant role in the relationship between cybervictimization and mental health disorders. Wright (Wright, 2024) argued that university students with a high level of family cohesion presented significantly decreased levels of depression and anxiety indicators. Likewise, Vieta-Piferrer et al. (Vieta-Piferrer et al., 2024) found that family support can serve as a resilience-based resource, weakening the relationship between cybervictimization and stress by mitigating the effects of social rejection and online degradation. Deep family ties can reverse the identity-threatening impacts of cybervictimization, lowering the probability of psychological failure, societal withdrawal, and continuing rumination (Barrera & Li, 1996). Additionally, family advice to ask for help from students’ counselors, colleagues, or other available university services might further block the acceleration of stress, anxiety, and depression symptoms. Therefore, the hypotheses below can be assumed:

**H4.**  *Family support can moderate the relationship between cybervictimization and stress.*

**H5.**  *Family support can moderate the relationship between cybervictimization and anxiety.*

**H6.**  *Family support can moderate the relationship between cybervictimization and depression.*

## 3. Methods

### 3.1. Materials

Data was collected using a self-structured survey scale designed primarily for this research. The “European Cyberbullying Intervention Project Questionnaire” (ECIPQ) introduced by Del Rey et al. (2015) was employed to measure cybervictimization (the victim side of cyberbullying). The scale has 11 items and measures students’ experiences of cybervictimization over the last two months, using a four-point scale, where 1 means never being subjected to cybervictimization and 4 means experiencing cybervictimization several times a week. Sample items include: “someone threatened me through messages”,” someone excluded me from online groups or conversations on purpose”. Furthermore, mental health was measured by three factors (stress, anxiety, and depression) derived from Lovibond and Lovibond’s shorter version of the DASS-21 scale (Lovibond & Lovibond, 1995). Each sub-scale has seven items reflecting stress, anxiety, and depression symptoms. Sample items include “I was unable to become enthusiastic about anything”, “I was worried about situations in which I might panic and make a fool of myself”, “I was intolerant of anything that kept me from getting on with what I was doing”. Participants were kindly asked to assess the level of agreement with each item using a 4-point Likert scale, where 0 indicated “no agreement” and 3 indicated “a high level of agreement”. Finally, family support was measured by four items derived from the “Multidimensional Scale of Perceived Social Support” (MSPSS) introduced by Zimet et al. (Zimet et al., 1988), which includes sample items such as “I get the emotional help & support I need from my family”. The employed scale was first assessed by 10 experts in academic life to confirm content validity and then pilot-tested with a small group of 20 students to ensure clarity and reliability. The final version of the questionnaire was circulated electronically via university official platforms or social network sites. 

### 3.2. Participants

A total of 790 higher education students freely contributed to the study. After eliminating incomplete replies, a total of 650 samples were retained for further analysis, yielding a response rate of 82%. This sample size exceeded the recommended thresholds for complex structural models in PLS-SEM, ensuring adequate statistical power to test both direct and moderating effects within the proposed model. Furthermore, we have implemented a sensitivity power analysis following Lakens (2022) employing a Monte Carlo simulation approach in R. With our sample size of 650, the analysis revealed that the model has adequate statistical power (≥0.80) to detect small-to-medium impact sizes for both direct and moderating effects in the suggested PLS-SEM model. These outcomes confirm that the study sample size is acceptable and adequate for further analysis using PLS-SEM.

The demographic statistics of the participants from KSA university students displayed a predictable university population. The majority of participants were undergraduate students (85%), with postgraduate students forming only 15%. Most respondents are within the normal university-age level between 18 and 25 years. Gender was quite balanced, with 49% male and 51% female, presenting equitable participation. Furthermore, different academic disciplines were represented, including business schools, medical sciences, education, and arts, which can strengthen the heterogeneity of the study sample and enhance the generalizability of the study’s results to the wider KSA higher education setting.

### 3.3. Procedures

This research paper used a cross-sectional approach to explore the psychological outcomes of cybervictimization and to test the key moderating role of family support among Saudi Arabian university students. This approach was selected due to its appropriateness for capturing the occurrence and immediate psychological consequences of cybervictimization within a large population at a single point in time, without the need for long-term tracking or experimental operations (Cummings, 2018), which could be unfeasible in this context.

### 3.4. Data Analysis Techniques

Data were analyzed using “Partial Least Squares Structural Equation Modeling” (PLS-SEM) in the SmartPLS program, given the exploratory nature of the current paper and the investigation of direct and moderating paths among latent unobserved variables (Joseph et al., 2022). PLS-SEM was chosen for its advantages in handling non-normally distributed data, its appropriateness for theory development, and its capacity to evaluate complex relationships involving multiple direct and moderator effects within a single model (J. F. Hair et al., 2021). The analysis ensued in two main stages: stage (1) evaluation of the measurement model (convergent validity, reliability, and discriminant validity) followed by stage (2) assessment of the structural model for hypothesis testing (Sarstedt et al., 2020). Bootstrapping options with 5000 iterations were conducted to estimate the standard errors and 95% confidence intervals for the path coefficients (β), including the moderating effects of family support on the relationship between cybervictimization and the three dimensions of mental health disorder. Bias-corrected and accelerated (BCa) confidence intervals were used to assess the statistical significance of the paths.

## 4. Study Results

### 4.1. Stage 1: Measurement Model Results

The reliability and validity of the provided measurement models were tested using PLS-SEM, as per the suggestions of (Sarstedt et al., 2022). J. F. Hair et al. (2021) introduced several main criteria to test the measurement models, including “composite reliability” (CR) and “average variance extracted” (AVE) for each latent variable. Table 1 illustrates the “factor loadings” (FL), C.R., “Cronbach’s α”, and the AVE. The measurement scale items’ reliability showed a reasonable result, with all items exceeding the suggested threshold of 0.5 (Tenenhaus et al., 2005). According to the emerged CR scores, all dimension values are above the lowest suggested score of 0.70 (J. F. Hair et al., 2021). Consequently, the model fulfilled the conditions for construct reliability. As per the scale’s convergent validity, the AVE values reported in Table 1 exceeded the suggested value of 0.50 (Leguina, 2015). To test discriminant validity, two more criteria were inspected (Chin, 2010), (1) the HTMT “heterotrait–monotrait ratio of correlations” (as shown in Table 2), which must be below the score of 0.90 (J. Hair & Alamer, 2022), and (2) the Fornell-Larcker criterion (as shown in Table 3), where all AVE square roots (diagonal numbers) should be above their intercorrelations with any other latent variables (below the diagonal number), confirming discriminant validity.

### 4.2. Stage 2: Inner Model Results and Hypothesis Testing

**Figure 1:**
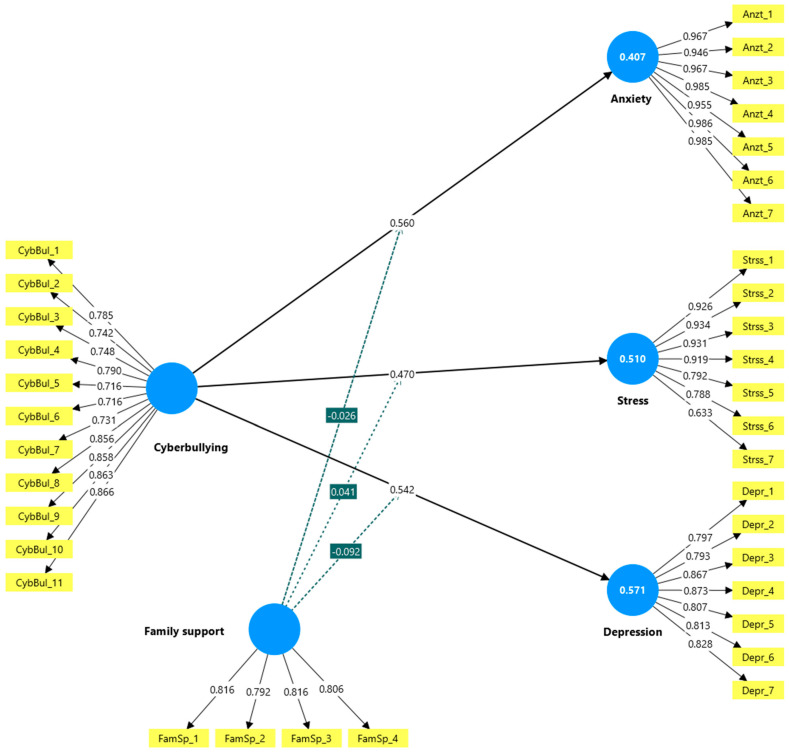
The research Model.

The structural inner model was inspected for predictive and explanatory capacity before testing the research hypotheses. This inspection was conducted using several criteria suggested by (Henseler & Sarstedt, 2013). First, the R^2^ “coefficient of determination “was visualized to assess the explanatory capabilities of the exogenous Variables in explaining the endogenous variables. As depicted in Figure 1, all dependent variables have a high value of R^2^ (anxiety: R^2^ = 0.407, stress: R^2^ = 0.510; depression: R^2^ = 0.571), demonstrating a high level of explanatory power. Second, the Q^2^ “predictive relevance” was determined employing the “Stone–Geisser test” via a blindfolding option. A Q^2^ value greater than 0.00 for dependent factors signifies that the model has adequate predictive power (anxiety: Q^2^ = 0.397, stress: Q^2^ = 0.499, depression: Q^2^ = 0.558). Furthermore, variable collinearity was evaluated by checking VIF scores to confirm that the multicollinearity between the study predictors stayed within the appropriate limits (VIF < 0. 5) (Daoud, 2017). As shown in Table 1, all VIF scores are satisfactory.

After safeguarding the predictive and explanatory power of the proposed model, the path coefficients (β) and their significant values (*p*) were inspected as depicted in Table 4 employing the bootstrapping option in PLS-SEM to evaluate the proposed hypotheses. The PLS-SEM report indicated that cyberbullying has a strong, positive, and significant impact on increasing anxiety symptoms among university students in Saudi Arabia (β = 0.560, t = 10.665, *p* < 0.001), supporting H1. Similarly, cyberbullying was found to have a strong, positive, and significant impact on depression (β = 0.542, t = 10.594, *p* < 0.001) and stress (β = 0.470, t = 9.965, *p* < 0.001), supporting H2 and H3.

**Figure 2:**
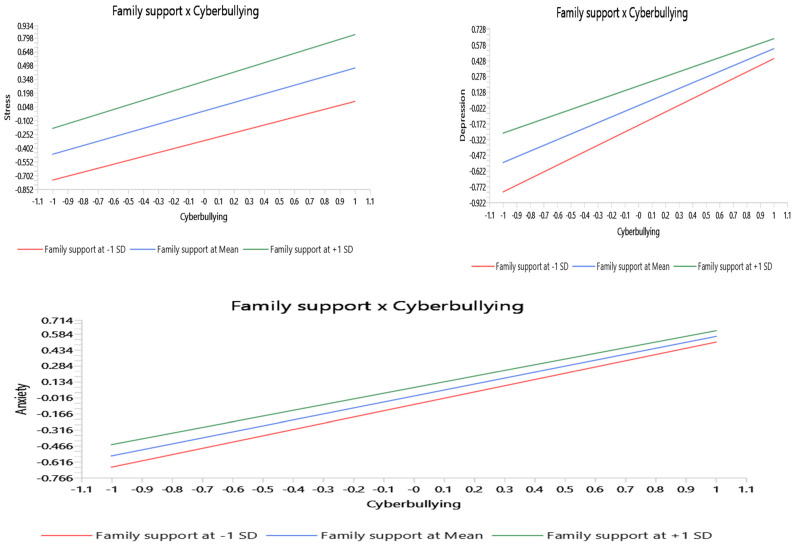
Simple slope.

Interestingly, as seen in Figure 2, the moderation effects results from the PLS-report revealed that family support has no significant moderating effect on the relationship between cyberbullying and anxiety (β = −0.026, t = 0.054, *p* = 0630), and cyberbullying and stress (β = 0.041, t = 0.843, *p* = 0.399), rejecting H4 and H6. However, family support was found to have a significantly negative moderating effect on the relationship between cyberbullying and depression (β = −0.092, t = 2.273, *p* < 0.5). This result revealed that family support failed to alleviate or mitigate the depressive symptoms resulting from cyberbullying among university students. Instead, the slope results in Figure 2 indicated a counterintuitive tendency whereby the higher family support level can cause a greater depressive reaction to cyberbullying. In other words, rather than acting as a buffering factor, family support seemed to increase the depressive impact of cybervictimization, rejecting H5.

## 5. Discussion and Implications

The results of PLS-SEM revealed that cyberbullying-victimization can significantly fuel the feeling of anxiety, stress, and depression among SA university students. These robust, positive and significant correlations indicate that cyberbullying-victimization is not simply a social concern but a dangerous psychological threat factor with considerable emotional outcomes. In the higher education settings, the correlations between cyberbullying-victimization and students’ well-being are more challenging as they regularly face academic pressure, social evolutions, and a higher level of digital involvement (Alharbi, 2022). Cyberbullying frequently happens anonymously and constantly, making it more emotionally destructive than conventional bullying (Kowalski et al., 2014). Students who experience cyberbullying-victimization are subjected to continued hypervigilance, predicting future threats, which can activate constant stress reactions and can directly fuel the feeling of anxiety (Chen et al., 2024). SA University students had one of the largest daily connection rates with social media usage (CST, 2023), which might increase the exposure to cyberbullying-victimization and intensify its psychological consequences (Backer & Awad, 2025). Furthermore, the SA cultural norms emphasize the importance of social image and family reputation, which might fuel the negative emotional outcomes of online provocation, leading to higher levels of anxiety (Alharbi, 2022).

The PLS-SEM results indicated that cyberbullying-victimization showed a significant positive correlation with stress, strengthening the emotional vulnerability generated by online threats. This outcome is consistent with the “Stress-Process Model” (Pearlin et al., 1981), which stated that continued exposure to uncontrollable stressors can fuel the adverse physiological and emotional effects. Some previous empirical evidence supported that the continuous exposure to cyberbullying victimization can elevate stress levels among adults (Pearlin et al., 1981; Zhu et al., 2024). The 24/7 connectivity of the digital world can diminish the boundaries that traditional communication methods provide to safeguard from stress, causing victims to be persistently exposed and constantly vigilant, and leading to interfering thoughts and emotional exhaustion (Kowalski et al., 2014). In a higher education environment, where academic pressures are significant, cyberbullying adds an extra emotional weight that can weaken concentration, minimize academic motivation, and foster burnout (Hutson, 2016). Accordingly, the significant relationship confirmed in this study is aligned with the global conclusion that cybervictimization is a dedicated predictor of persistent stress among higher education students.

The results also indicated that cybervictimization can significantly increase the feeling of depression among KSA students, confirming previous evidence that online victimization is one of the main predictors of depressive symptoms. The “Cognitive Model of Depression” (Beck, 1967) argued that adverse interpersonal conditions can activate the maladaptive cognitive behavior, such as blaming oneself and learned vulnerability, all of which are well-known outcomes of cyberbullying (Vieta-Piferrer et al., 2024). Previous evidence in diverse cultural settings demonstrated that cyberbullying is regularly correlated with misery, depression, decreased self-worth, and psychological withdrawal (Piotrowski & Watt, 2024). The lasting and enduring nature of digital harassment increases depressive responses, as victims might feel degraded in front of large public audiences and be unable to avoid the digital tracks of violence (Barlett et al., 2021). Moreover, results from SA and the wider MENA countries showed that cyberbullying can contribute to increased depression symptoms and decreased overall life wellbeing among higher education students (Al-Amer et al., 2025).

The moderation analysis produced a notable and somewhat unexpected pattern where family support did not significantly moderate the relationship between cyberbullying victimization and either stress or anxiety, leading to the rejection of H4 and H6. Despite the well-established buffering role of family support in the psychological literature (Cohen & Wills, 1985), these findings suggest that, within the context of Saudi Arabian university students, family support alone may be insufficient to offset the emotional and psychological harms caused by cyberbullying. Because of the complex digital nature of cyberbullying, families might lack the digital literacy or deep understanding required to offer helpful coping approaches (Aboujaoude et al., 2015). This might help in explaining why family support failed to significantly minimize stress and anxiety outcomes of cyberbullying victimization in this paper. The SA cultural settings add a further layer of understanding. Family hierarchy in SA society is collectivistic with strong bonds; therefore, family members (i.e., students) frequently hesitated to reveal negative experiences concerning shame, image, or social humiliation, which are main aspects of cybervictimization (Alharbi, 2022). Research in similar SA cultures (Middle Eastern region) revealed that adults tend to avoid discussing sensitive subjects with their parents due to a persistent fear of overreaction, preventive behavioural controls, or escalation to university-level authorities (Almakrob & Alduais, 2025).

The results revealed that support from family had a negative moderating influence on the path from cyberbullying-victimization to depression as a mental health disorder. This result indicated that higher levels of support from family were related to higher levels of depression responses, which means a reverse-buffering impact. This result, surprisingly, contradicted the established stress-protective assumption, which holds that family and social support regularly moderate the adverse psychological effects of stressful conditions (Cohen & Wills, 1985). Several justifications may be responsible for these paradoxical results. First, higher levels of family support and involvement may develop into overprotective issues, which might weaken students’ coping ability and increase their depressive responses (Rafaeli & Gleason, 2009). University students in SA who are subjected to cyberbullying-victimization might accordingly perceive extra pressure, a sense of guilt, or even shame when they feel that their struggles negatively affect their family’s reputation. Second, previous research declared that people with high family engagement regularly respond more severely to cybervictimization, as such bad experiences damage social safety (Wright, 2015). Third, cultural norms and values might play a significant role. In collectivist societies, family relationships are characterized by high levels of emotional mutuality, strong beliefs in harmony, and high parental involvement in daily life. While this can fuel strong ties, it can also upsurge emotional pressure, generating a context where adverse experiences such as cyberbullying victimization induce intensified distress levels (Dwairy & Achoui, 2010). Accordingly, support from family, rather than providing a protective tool, may unconsciously strengthen adults’ emotional effects to cybervictimization.

The results of the current study have some significant theoretical and practical implications. The significant correlations between cyberbullying-victimization and stress, anxiety, and depression can strengthen the conceptualization of cyberbullying-victimization as a major emotional stressor. This assumption extends the established frameworks developed by “Beck’s Cognitive Model” and the “Stress-Process Model” to digital settings. The study’s findings also challenged the widely held assumption about the protective role of family support. While the traditional established stress-buffering assumption argued that support from family members regularly alleviates the negative impacts of stressful experiences, the present study indicated that family support does not mitigate the influences of cyberbullying victimization on stress and anxiety, nor safeguards against depressive signs. Alternatively, the reverse-buffering influence inspected for depression indicates that, in certain cultural settings (i.e., SA), support from family members may inadvertently strengthen psychological distress. This assumption highlighted the need to incorporate concepts such as interfering or incompatible support into existing social support frameworks, particularly within collectivist cultures. With regard to the practical implications, the current results underscore the critical necessity for university leaders to prioritize cyberbullying-victimization as a key mental health issue. University help centers should integrate routine screening for exposure to cyberbullying and design customized interventions that equip university students with digital resilience and coping strategies. Considering that support from family members may be insufficient on their own, universities should also consider improving colleague-based support communities, as colleagues often act as a major source of psychological validation in emerging adulthood.

## 6. Limitations and Future Research Opportunities

Several limitations can be acknowledged and can be employed as directions for further research opportunities. First, the cross-sectional approach might limit the ability to assume causal interactions between cyberbullying-victimization and mental health consequences. Therefore, a longitudinal design is required to test how the supposed relationships advance over time and whether cyberbullying-victimization can cause sustained mental health outcomes among KSA higher education students. Second, this study focused mainly on SA higher education students, which may limit the generalizability of the results to other settings. Cross-cultural research would be beneficial for acknowledging the universal aspects of cybervictimization and family support. Third, the study tested family support as a major moderating variable; however, other moderating variables, such as colleagues’ support and university service support, were not tested. Considering that family support either showed no moderating effect or even presented a counterintuitive reverse-alleviating impact for depressive symptoms, future research should test more aspects of social support, as well as other mediating or moderating factors that might be more effective at alleviating the psychological outcomes of cyberbullying-victimization.

## Figures and Tables

**Table 1:** Psychometric statistics of the study scale.

Factors	Items	FL	*a*	CR	AVE	VIF
Anxiety			0.910	0.911	0.941	
	Anzt_1	0.967				1.879
	Anzt_2	0.946				2.301
	Anzt_3	0.967				2.155
	Anzt_4	0.985				2.085
	Anzt_5	0.955				3.898
	Anzt_6	0.986				2.150
	Anzt_7	0.985				2.316
Cyberbullying			0.939	0.941	0.625	
	CybBul_1	0.785				1.447
	CybBul_10	0.863				2.988
	CybBul_11	0.866				2.080
	CybBul_2	0.742				3.601
	CybBul_3	0.748				2.175
	CybBul_4	0.790				3.899
	CybBul_5	0.716				2.066
	CybBul_6	0.716				1.827
	CybBul_7	0.731				2.494
	CybBul_8	0.856				1.455
	CybBul_9	0.858				1.008
Depression			0.922	0.922	0.682	
	Depr_1	0.797				1.849
	Depr_2	0.793				2.854
	Depr_3	0.867				2.861
	Depr_4	0.873				2.699
	Depr_5	0.807				3.306
	Depr_6	0.813				3.865
	Depr_7	0.828				1.347
Family Support			0.823	0.825	0.652	
	FamSp_1	0.816				1.766
	FamSp_2	0.792				1.748
	FamSp_3	0.816				1.757
	FamSp_4	0.806				1.655
Stress			0.934	0.937	0.727	
	Strss_1	0.926				3.655
	Strss_2	0.934				1.344
	Strss_3	0.931				3.959
	Strss_4	0.919				3.093
	Strss_5	0.792				3.575
	Strss_6	0.788				3.227
	Strss_7	0.633				1.441

**Table 2:** Heterotrait-monotrait ratio (HTMT).

	Anxiety	Cyberbullying	Depression	Family Support	Stress
Anxiety					
Cyberbullying	0.653				
Depression	0.484	0.791			
Family support	0.556	0.729	0.737		
Stress	0.478	0.733	0.640	0.731	

**Table 3:** Fornell-Larcker criterion.

	Anxiety	Cyberbullying	Depression	Family Support	Stress
Anxiety	0.970				
Cyberbullying	0.635	0.791			
Depression	0.465	0.637	0.826		
Family support	0.508	0.635	0.645	0.808	
Stress	0.458	0.682	0.590	0.642	0.853

**Table 4:** Research hypotheses results.

	β	*T* Statistics	*p* Values	Results
Cyberbullying -> Anxiety	0.560	10.665	0.000	H1-Supported
Cyberbullying -> Depression	0.542	10.594	0.000	H2-Supported
Cyberbullying -> Stress	0.470	9.695	0.000	H3-Supported
Moderation Paths				
Family support x Cyberbullying -> Anxiety	−0.026	0.481	0.630	H4-Rejected
Family support x Cyberbullying -> Depression	−0.092	2.273	0.023	H5-Rejected
Family support x Cyberbullying -> Stress	0.041	0.843	0.399	H6-Rejected

## Data Availability

Data available on request from corresponding author due to privacy/ethical restrictions.
